# Efficacy of Intravenous Paracetamol in Combination with Lidocaine Pretreatment for Reducing Pain During Injection of Propofol

**DOI:** 10.7759/cureus.6926

**Published:** 2020-02-09

**Authors:** Muhammad Hayat, Gauhar Afshan, Muhammad Nasir, Samie Asghar, Abdul Monem

**Affiliations:** 1 Anaesthesiology, Northwest General Hospital & Research Centre, Peshawar, PAK; 2 Anaesthesiology, The Aga Khan University, Karachi, PAK; 3 Anaesthesiology, The Aga Khan Univeristy, Karachi, PAK

**Keywords:** pain, injection, propofol, paracetamol, lidocaine, analgesia, pain management

## Abstract

Introduction

The association of pain and discomfort of moderate to high severity and a high incidence with the intravenous (IV) administration of propofol is well known. Various physical and pharmacological methods are used to minimize propofol-induced pain, but the best intervention is still unknown. Therefore, our aim was to determine the analgesic efficacy of IV paracetamol when used in combination with lidocaine pretreatment in reducing propofol injection pain.

Materials and methods

This double-blind, randomized controlled trial was conducted after receiving the approval of our institutional research ethics board. A total of 74 patients were included after providing informed consent, and participants were placed into two equal groups: group A received IV paracetamol (1 g) in combination with lidocaine pretreatment prior to the injection of propofol, and group B received lidocaine pretreatment alone prior to propofol injection. After propofol injection, all participants were asked to evaluate pain on the visual analog scale.

Results

Patients who received the lidocaine-paracetamol combination reported significantly more pain-free responses (51.35%) than those from patients who received lidocaine pretreatment alone (8.11%; P<0.05). The analgesic efficacy of group A was positive in 36 patients (97.3%), and for group B, the analgesic efficacy was positive in 24 patients (64.9%).

Conclusion

The administration of IV paracetamol with lidocaine pretreatment was more effective than lidocaine pretreatment alone in reducing the pain caused by the injection of propofol. Physicians should consider using IV paracetamol in combination with lidocaine pretreatment when patients require IV propofol to ease patient suffering and reduce pain, which may help provide optimal patient care.

## Introduction

Propofol is a popular intravenous (IV) anesthetic induction agent that causes pain during administration. The incidence of propofol-induced pain is 28% to 90% in patients receiving propofol alone with no other intervention or medication [1]. The exact mechanism of pain due to IV propofol is unknown. Different physical and pharmacological strategies have been assessed as means to reduce propofol-associated pain, but the best means has yet to be determined [2-4]. Propofol-induced pain on administration may prevent the use of propofol anesthesia in pediatric patients [5]. Moreover, propofol-associated pain further adds to the general pain load of already stressed patients.

Paracetamol is a synthetic, non-opiate, centrally acting analgesic derived from p-aminophenol. It is widely used for pain management preoperatively as co-analgesia [6,7]. The analgesic effect of paracetamol is most likely due to its effect on the central nervous system, although paracetamol has also shown some peripheral or local effects [5]. A variant of the cyclooxygenase-2 enzyme has been discovered that is sensitive to paracetamol. According to a previous study, the incidence of pain for a patient receiving lidocaine was 20%, and the incidence of pain in patients receiving IV paracetamol with lidocaine was 2% [8]. Recently published data indicate that pain reduction can be more effectively achieved by using more than one means of pain reduction rather than using a single agent of pain reduction [9]. Therefore, we wanted to assess the use of both lidocaine pretreatment and IV paracetamol for reducing propofol-associated injection pain. The purpose of the study was to determine whether a single IV dose of paracetamol given 30 minutes prior to propofol injection can reduce propofol injection pain. Therefore, we evaluated the analgesic efficacy of IV paracetamol when used in combination with lidocaine pretreatment in reducing propofol injection pain.

## Materials and methods

This double-blind, randomized controlled trial was conducted in the operating rooms of a tertiary care center after approval from the institutional ethical review committee. The study included patients aged 18 to 60 years and in the American Society of Anesthesiologists (ASA) status I and ASA II, who were admitted for elective surgery under general anesthesia after providing informed consent to participate. Patients who provided consent were selected via a nonprobability consecutive sampling technique. Any patients taking other analgesics preoperatively, those with known allergies to any drug used in the study, those with deranged hepatic functions, those with ASA III or higher status, those with a language barrier, those unable to understand the visual analog scale (VAS), and any pregnant women were excluded from the study. A unique randomization number was allotted to each participant, who was randomly assigned into two equal groups of 34 patients. Group A patients received IV paracetamol (1 g) with lidocaine pretreatment, and group B served as the control group and received lidocaine pretreatment alone. The study used 74 similar looking bottles labeled as “study drug” with the randomization number for masking the contents from the primary investigators and the patients. The enrolled patients were taught how to use the 100-point VAS to rate their pain levels. All patients were premedicated with midazolam one hour before surgery. In the preoperative area, after standard monitoring, the study drug (0.9% saline or paracetamol) was infused over 15 to 20 minutes under the observation of the primary anesthetist. Afterward, the patients were moved to the operating room. After a pneumatic tourniquet was applied, the vein which would be used for propofol injection was pretreated with 2 mL of 2% lidocaine. After one minute of pretreatment, the tourniquet was released and one-fourth of the calculated dose of propofol was injected intravenously over five seconds by an electronic pump. After injection, the patient was asked whether he or she felt pain, and the pain score was marked according to the 100-point VAS. Pain severity levels were defined as VAS 0 for no pain, VAS 1 to 30 for mild pain, VAS 31 to 70 for moderate pain, and VAS >70 for severe pain. Following that measurement, the complete anesthetic dose of propofol was administered and anesthesia continued as per the plan.

Data analysis

All statistical procedures were performed using IBM SPSS Statistics for Windows, Version 19.0. (Armonk, NY: IBM Corp.). We computed the mean and standard deviation for all quantitative variables including age, weight, and height, while qualitative data like sex, pain, and efficacy of paracetamol were calculated in frequency and percentage. Chi-square and Fisher’s exact tests were applied to compare the proportion of pain between the two groups. P-value <0.05 was considered to be significant. Stratification was done to control effect modifiers like age, sex, height, and weight to observe an outcome (pain).

## Results

A total of 74 patients were enrolled in the study (36 men [48.6%] and 38 women [51.35%]). The study population age, weight, height, and hemodynamic characteristics are presented in Table 1. The sex distribution among groups was also similar; group A: 45.95% men, 54.05% women, and group B: 51.35% men, 48.65% women.

**Table 1:** Comparison of demographic and hemodynamic characteristics between groups BP, blood pressure

Demographic Characteristics	Group A (n=37)	Group B (n=37)	P-Values
Age (years)	36.62±11.56	37.70±11.34	0.68
Weight (kg)	68.97±11.11	70.04±8.32	0.53
Height (cm)	162.46±8.94	163.76±8.24	0.64
Systolic BP (mmHg)			
Pre	122.73±10.13	131.59±8.96	0.06
Post	119.59±8.85	128.14±9.71	0.04
Diastolic BP (mmHg)			
Pre	77.03±5.11	77.46±5.69	0.0005
Post	75.32±4.95	77.41±5.27	0.0005
Heart rate (beat/min)			
Pre	80.41±13.34	85.84±11.33	0.73
Post	81.78±12.64	87.70±11.48	0.08

The incidence of pain in the control group (group B) was higher than group A. In group B, 34 patients (91.89%) reported pain on injection, while only 18 patients (48.64%) in group A reported pain on injection. For pain severity, four patients in group B (10.81%) and no patients in group A reported severe pain on injection. One patient in group A (2.7%) reported moderate pain, while nine patients in group B (24.32%) reported moderate pain. A total of 17 patients in group A (45.95%) reported mild pain and 21 patients in group B (56.76%) reported mild pain. Nineteen patients in group A reported no pain on injection (51.35%), while only three patients in group B (8.11%) reported no pain on injection. The assessment of analgesic efficacy, defined as positive (“YES”) for VAS score of 0 to 30 and negative (“NO”) for VAS scores of 31 to 100 for each group is shown in Table 2.

**Table 2:** Comparison of analgesic efficacy between groups Chi-square value = 12.68

Final Outcome (Analgesic Efficacy)	Group A (n=37)	Group B (n=37)	P-Values
Yes	36 (97.3%)	24 (64.9%)	0.0005
No	1 (2.7%)	13 (35.1%)	

No untoward effects related to paracetamol were found in patients in group A. There was no significant difference in analgesic efficacy in groups A and B with respect to sex and height (P<0.05). Analgesic efficacy results in each group with respect to age and weight are described in Table 3.

**Table 3:** Comparison of analgesic efficacy between groups †Fisher’s exact test because two cell have expected count <2

Stratification	Final Outcome (Analgesic Efficacy)	Group A (n=37)	Group B (n=37)	P-Values
Age of the patients (years)				
≤40 years	Yes	25 (100%)	15 (71.4%)	0.006†
	No	0 (0%)	6 (28.6%)	
	Total	25	21	
>40 years	Yes	11 (91.7%)	9 (56.3%)	0.048†
	No	1 (8.3%)	7 (43.8%)	
	Total	12	16	
Weight of the patients (kg)				
≤70 kg	Yes	18 (100%)	12 (60%)	0.003†
	No	0 (0%)	8 (40%)	
	Total	18	20	
>70 kg	Yes	18 (94.7%)	12 (70.6%)	0.067†
	No	1 (5.3%)	5 (29.4%	
	Total	19	17	

## Discussion

Propofol has a fast onset, smooth anesthesia induction, favorable effect on upper airway reflexes, a smooth and rapid recovery, and minimal association with postoperative nausea and vomiting, making it a popular IV anesthetic induction agent [10,11]. However, one of the complications of propofol is that it causes pain on IV injection due to its highly osmotic lipid solvent and propofol’s irritant effect on the vascular intima [12].

Canbay et al. studied the effect of 50 mg paracetamol pretreatment on non-premedicated (with sedatives or analgesics) patients and concluded that paracetamol pretreatment was an effective intervention to reduce propofol injection pain [8]. Ozkan et al. studied the effect of 100 mg paracetamol pretreatment with and without tourniquet in non-premedicated (with sedatives or analgesics) patients for the prevention of propofol-induced pain and concluded that paracetamol pretreatment using a tourniquet has equivalent efficacy as lidocaine pretreatment regarding propofol injection pain [13]. In that study, pain was assessed by a four-digit numeric rating scale of 0 to 3 for no pain, mild, moderate, and severe pain, respectively. None of the patients reported moderate or severe pain on a one-fourth dose of propofol injection in the tourniquet group. Our study found that no patients in group A had severe pain, and only one (2.70%) patient reported moderate pain with paracetamol with lidocaine pretreatment.

Dedic et al. studied the effect of oral paracetamol (1 g) on prevention of propofol pain using the VAS scoring system; they found that no patients reported severe pain, 75 (72.81%) reported no pain, and 28 (27.19%) reported mild, bearable pain on propofol injection [14]. However, it should be noted that Dedic et al. used diclofenac 50 mg, midazolam 7.5 mg, and fentanyl 2.5 U/kg before the propofol injection, where propofol was premixed with lidocaine (1 mg for 1 mL of propofol). This is a much higher reduction than our study findings (51.35% pain free), which might be related to the additional use of diclofenac 50 mg and fentanyl 2.5 U/kg before propofol injection. El-Radaideh studied the effect of 40 mg paracetamol pretreatment on propofol injection pain and concluded that 54% of the patients were pain free [15]. Paracetamol has been used for propofol injection pain in doses ranging from as low as 40 mg to 1 g. However, we found that 1 g of IV paracetamol in combination with lidocaine pretreatment is superior to lidocaine pretreatment alone in reducing pain associated with propofol administration.

The strength of this study is that it is a randomized, double-blind controlled trial, and all the cases were performed by one anesthetist to eliminate observer bias. Also, it was a therapeutic study in which patients benefited. To our knowledge and according to the available literature, this study is the first of its kind in our population. One potential weakness of the study is the sample size; a larger size may have added further support to our findings.

## Conclusions

The administration of IV paracetamol in combination with lidocaine for pretreatment before propofol injection is a superior method to lidocaine pretreatment alone in reducing pain caused by propofol injection. For patients who require propofol during their care, physicians should consider using IV paracetamol in combination with lidocaine pretreatment, when appropriate, to ease patient suffering and reduce pain, which may help provide optimal patient care.

## References

[1] Zahedi H, Nikooseresht M, Seifrabie M (2009). Prevention of propofol injection pain with small-dose ketamine. Middle East J Anesthesiol.

[2] Lembert N, Wodey E, Geslot D, Ecoffey C (2002). Prevention of pain on injection with propofol in children: comparison of nitrous oxide with lidocaine. Ann Fr Anesth Reanim.

[3] Picard P, Tramer MR (2000). Prevention of pain on injection with propofol: a quantitative systematic review. Anesth Analg.

[4] Euasobhon P, Dej-Arkom S, Siriussawakul A (2016). Lidocaine for reducing propofol-induced pain on induction of anaesthesia in adults. Cochrane Database Syst Rev.

[5] Borazan H, Erdem TB, Kececioglu M, Otelcioglu S (2010). Prevention of pain on injection of propofol: a comparison of lidocaine with different doses of paracetamol. Eur J Anaesthesiol.

[6] Moon YE, Lee YK, Lee J, Moon DE (2011). The effects of preoperative intravenous paracetamol in patients undergoing abdominal hysterectomy. Arch Gynecol Obstet.

[7] Afhami MR, Hassanzadeh P, Panahea JR (2007). Pre-emptive analgesia with Paracetamol in postoperative pain. Rawal Med J.

[8] Canbay O, Celebi N, Arun O, Karagoz AH, Saricaoglu F, Ozgen S (2008). Efficacy of intravenous acetaminophen and lidocaine on propofol injection pain. Br J Anaesth.

[9] Kwak HJ, Min SK, Kim JS, Kim JY (2009). Prevention of propofol-induced pain in children: combination of alfentanil and lidocaine vs alfentanil or lidocaine alone. Br J Anaesth.

[10] Srivastava U, Mishra AR, Sharma S (2008). Anaesthesia for direct laryngoscopy with propofol and fentanyl or sufentanil. Indian J Otolaryngol Head Neck Surg.

[11] Gauger PG, Shanks A, Morris M, Greenfield ML, Burney RE, O'Reilly M (2008). Propofol decreases early postoperative nausea and vomiting in patients undergoing thyroid and parathyroid operations. World J Surg.

[12] Jung JA, Choi BM, Cho SH (2010). Effectiveness, safety, and pharmacokinetic and pharmacodynamic characteristics of microemulsion propofol in patients undergoing elective surgery under total intravenous anaesthesia. Br J Anaesth.

[13] Ozkan S, Sen H, Sizlan A, Yanarates O, Mutlu M, Dagli G (2011). Comparison of acetaminophen (with or without tourniquet) and lidocaine in propofol injection pain. Klinik Psikofarmakol Bülteni.

[14] Dedic A, Adam S, Gommers D, Van Bommel J (2010). Propofol injection pain-is it still an issue? The effect of premedication. Minerva Anestesiol.

[15] El-Radaideh KM (2007). Effect of pretreatment with lidocaine, intravenous paracetamol and lidocaine-fentanyl on propofol injection pain: comparative study. Rev Bras Anestesiol.

